# Repurposing Itraconazole in Combination with Chemotherapy and Immune Checkpoint Inhibitor for Cancer

**DOI:** 10.3390/medsci14010055

**Published:** 2026-01-22

**Authors:** Camille E. Zonfa, Anita Thyagarajan, Ravi P. Sahu

**Affiliations:** 1 Boonshoft School of Medicine, Wright State University, Dayton, OH 45435, USA; zonfa.2@wright.edu; 2 Department of Pharmacology and Toxicology, Boonshoft School of Medicine, Wright State University, Dayton, OH 45435, USA; anita.thyagarajan@wright.edu

**Keywords:** drug repurposing, itraconazole, immune checkpoint inhibitors, combination therapy, cancer immunotherapy, immune modulation

## Abstract

Cancer remains a significant global health burden despite advances in diagnosis and treatment. In recent years, drug repurposing has emerged as a promising strategy in oncology, offering reduced costs and shorter development timelines compared with de novo drug discovery. Among repurposed agents, the antifungal drug itraconazole has demonstrated anticancer activity across multiple tumor types, particularly when used in combination with other therapeutic modalities. In this review, we summarize current preclinical and clinical evidence supporting the use of itraconazole in cancer therapy, with a specific focus on its combination with chemotherapeutic agents and programmed cell death protein 1 (PD-1) immune checkpoint inhibitors. We highlight proposed mechanisms underlying this synergy, including modulation of tumor metabolism, angiogenesis, and immune signaling pathways. Additionally, we discuss key challenges and limitations, such as drug–drug interactions and toxicity considerations, that must be addressed to optimize clinical translation. Overall, the combination of itraconazole with chemotherapy or anti-PD-1 therapy represents a promising therapeutic strategy warranting further investigation in well-designed trials.

## 1. Introduction

Cancer remains a leading global health concern, accounting for nearly one in six deaths worldwide [1,2]. According to the American Cancer Society, there are estimated to be two million new cancer cases and 611,720 cancer deaths in the United States in 2024 alone. The most common types include lung, breast, colorectal, and prostate cancer, which continue to challenge healthcare systems with their high incidence and treatment complexity [1,3]. Despite significant advancements in diagnosis and treatment, many patients continue to experience limited responses and a multitude of side effects while undergoing conventional therapies, underscoring the need for innovative and more effective treatment strategies [4,5].

In recent years, drug repurposing has gained traction as a promising approach in oncology. Drug repurposing is the practice of using existing medications developed for non-cancer indications to treat malignancies. This strategy offers several advantages: it leverages known safety profiles, accelerates the drug development process, and reduces costs [5]. One such candidate, itraconazole, an antifungal agent commonly used to treat systemic fungal infections, has shown potential anticancer activity in preclinical studies and early clinical data [6,7,8]. Preclinical data have demonstrated that itraconazole can inhibit cancer cell proliferation, suppress angiogenesis, and modulate autophagy [9,10,11].

Given the importance of the host immune system in regulating tumor growth as well as factors affecting the anti-tumor immune response of therapeutic agents, significant efforts have been dedicated towards understanding the insights of tumor immune regulation, such as the involvement of immune checkpoints (ICs) [12,13,14]. To that end, strategies to block ICs, such as programmed cell death protein 1 (PD-1) receptor interaction with its ligand, PD-L1, with immune checkpoint inhibitors, have revolutionized cancer treatment [15,16]. While immune checkpoint inhibitors like pembrolizumab and nivolumab that block the PD-1 receptor have shown considerable success in treating malignant tumors in various cancer lines, the therapeutic effect is still unsatisfactory for many patients [17,18]. Patients either exhibit primary resistance to immune checkpoint inhibitors or develop resistance over time. The complexity of the tumor microenvironment (TME), including immunosuppressive cells, abnormal vasculature, and metabolic barriers, can hinder the immune system’s ability to attack cancer cells effectively [18]. Therefore, it is necessary to develop strategies to enhance the immunotherapy effects of anti-PD-1 drugs [18]. Combining anti-PD-1 therapy with drugs like itraconazole may help overcome these challenges by modulating the tumor microenvironment and enhancing immune activity.

Similar resistance mechanisms also undermine the efficacy of traditional chemotherapy. The taxane-platinum chemotherapeutic regimen remains a cornerstone of cancer therapy today, despite being introduced over four decades ago. Taxanes and platinum agents induce cancer cell death primarily through the disruption of mitosis and DNA damage-mediated apoptosis, respectively. Despite their clinical importance, the effectiveness of this regimen has been limited by the development of drug resistance and significant treatment-related toxicities. The addition of itraconazole to chemotherapy has emerged as a potential strategy to overcome resistance mechanisms and improve therapeutic outcomes [19].

By examining the mechanisms, clinical evidence, and challenges associated with this combination therapy, this paper highlights how integrating repurposed drugs with chemotherapy or immunotherapy could improve outcomes and expand treatment options for cancer patients. Ultimately, the goal is to evaluate whether such a combination strategy holds meaningful clinical promise and could contribute to addressing the limitations of current cancer therapies.

## 2. Drug Repurposing in Cancer Therapy

Traditionally, drug development is a time-consuming process that includes identifying the therapeutic drug molecule that effectively treats a disease [5]. There are five stages to the drug development process, causing the entire process to take roughly 10–17 years and cost millions of dollars [5,20,21]. Additionally, de novo drug development is associated with high rates of failure, with nearly 90% of candidate molecules ultimately rejected due to unexpected safety and efficacy concerns [5,22]. Due to the challenges associated with de novo drug development, researchers have been exploring the impact of drug repurposing on the efficacy of standard-of-care treatments for cancer.

Drug repurposing involves the exploration of already Food and Drug Administration (FDA)-approved and clinically used drugs for new therapeutic purposes in the treatment of other diseases and illnesses. The concept of drug repurposing has improved the success rate of medication development, as repurposed drugs have an established safety profile and have already been extensively studied [5,23,24]. Along with this advantage, the utilization of drug repurposing has a significantly shorter development process and is associated with lower costs [5,25]. This is especially important in oncology, where cancer therapies are increasingly expensive yet frequently provide suboptimal results and only slight improvements in patient survival [26,27]. Drug repurposing has the potential to make cancer treatment more accessible, reduce the financial burden on patients, and address the unsustainable costs of cancer treatment [5,25,26]. With their numerous advantages, a large portion of newly approved drugs in the United States are repurposed drugs, and recent efforts have increasingly centered on cancer [5,28].

Given the global burden of cancer and the urgent need for novel treatment strategies, drug repurposing has quickly become an important aspect of cancer therapy [5,29]. Many FDA-approved drugs developed for non-oncologic conditions exhibit anticancer properties because they target fundamental cellular pathways that are also involved in tumor development [5,30]. These pathways are closely related to the eight hallmarks of cancer, including the capability to sustain proliferative signaling, evade growth suppressors, resist cell death, enable replicative immortality, induce and access vasculature, activate invasion and metastasis, reprogram cellular metabolism, and avoid immune destruction [31]. As these mechanisms are not unique to cancer cells, drugs developed for other diseases can often influence tumor biology through overlapping signaling cascades [30,31,32,33].

Drug repurposing may also help overcome the persistent problem of drug resistance in cancer therapy, which can be intrinsic or acquired [31,34]. This phenomenon is one of the most formidable obstacles in oncology and is deeply rooted in the biological adaptability of cancer cells. As described in the hallmarks of cancer, tumor cells possess a remarkable ability to evolve, utilizing genetic and epigenetic changes to bypass therapeutic effects [31]. Resistance mechanisms include increased drug efflux, enhanced DNA repair, mutations in drug targets, and activation of compensatory signaling pathways [34,35]. These adaptations can render even the most advanced chemotherapeutic, radiologic, and targeted therapies ineffective over time [35]. Drug repurposing expands the arsenal of therapeutic strategies and allows researchers to target cancer cells in novel ways, especially pathways not traditionally associated with cancer therapy [5,25,35]. By disrupting multiple facets of tumor biology, repurposed agents may help circumvent resistance mechanisms and restore treatment efficacy in otherwise refractory cases [5,25].

Several drugs have been successfully repurposed for oncology. Ritonavir, originally developed as an antiviral for HIV (human immunodeficiency virus), has demonstrated antitumor effects by inducing apoptosis and inhibiting malignant progression in breast, pancreatic, and ovarian cancers, as well as showing synergy with other anticancer agents [30,36]. Similarly, metformin, commonly used for type II diabetes, has been shown to have anticancer properties through inhibition of mammalian target of rapamycin (mTOR) activity, leading to the prevention of protein synthesis and cell growth [37,38]. These examples highlight how drugs originally designed for entirely different indications can possess mechanisms that interfere with cancer progression.

## 3. Repurposed Antifungals: Clinical Use and Mechanisms of Action

Along similar lines, antifungals have been explored as potential repurposed drugs for cancer treatment. The commonly used antifungals, including itraconazole, fluconazole, miconazole, ketoconazole, voriconazole, posaconazole, isavuconazole, and oteseconazole, have a crucial role in the treatment of fungal infections [39]. Of note, ergosterol synthesis inhibitors are a class of antifungal medications and are commonly called “azoles” [39,40]. Ergosterol is an important component of fungal membranes, serving the same role as cholesterol in human cell membranes. Since ergosterol is not present in human cells, it is an important target for antifungal drugs. The azoles inhibit lanosterol 14-alpha-demethylase, the enzyme that converts lanosterol to ergosterol [39]. This inhibition of a cell membrane component increases the permeability of fungal cell membranes, leading to cell lysis and death. The azole antifungals have demonstrated potent antifungal properties and have fewer adverse effects than other antifungal drugs [39].

**Figure 1:**
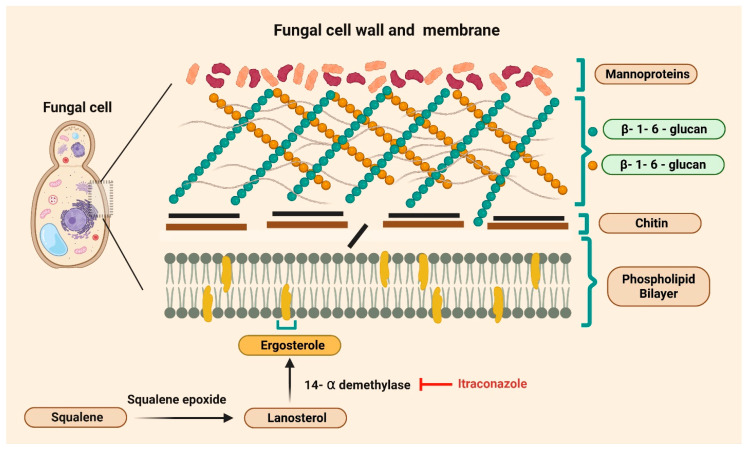
Mechanisms of itraconazole function. The fungal cell wall consists of mannoproteins, β-1,6-glucan, chitin, and a phospholipid bilayer containing ergosterol. Squalene is converted to lanosterol by the enzyme squalene epoxidase, after which 14-α-demethylase converts lanosterol into ergosterol. Itraconazole inhibits 14-α-demethylase, thereby blocking ergosterol synthesis.

Itraconazole, one of the “azole” drugs, was developed in the late 1980s as a first-generation triazole antifungal designed to improve potency, spectrum of activity, and tolerability compared with earlier azoles such as ketoconazole [41]. Itraconazole is traditionally used to treat a broad range of fungal infections, including blastomycosis, histoplasmosis, and aspergillosis [40]. It is also commonly used for chronic pulmonary aspergillosis, onychomycosis, and other superficial and systemic fungal infections in both immunocompetent and immunocompromised patients [40]. Itraconazole shares the same mechanism as the other “azoles,” exerting its antifungal effects through inhibiting the synthesis of ergosterol [39]. The schematic representation of itraconazole’s mechanisms is shown in Figure 1. Additionally, itraconazole can be used as prophylaxis in those at risk of systemic fungal infections, such as immunocompromised patients [40]. As an FDA-approved drug with a well-established safety profile, itraconazole has been widely prescribed for both superficial and systemic mycoses [40,42,43].

## 4. Experimental and Clinical Studies

### 4.1. Experimental Studies

Beyond its antifungal applications, itraconazole has demonstrated significant anticancer activity across a variety of tumor types in preclinical and clinical studies. Itraconazole’s anticancer activity has been extensively studied compared to other “azole” drugs [11,44,45,46,47,48,49]. Literature supports its efficacy in cancers such as basal cell carcinoma, colon cancer, non-small cell lung cancer (NSCLC), prostate cancer, and ovarian cancer [7,11,45,46,48,50]. Additionally, itraconazole has been shown to suppress tumor angiogenesis by inhibiting vascular endothelial growth factor receptor 2 (VEGFR2) glycosylation and trafficking, thereby reducing blood vessel formation critical for tumor growth [11,51]. Itraconazole also modulates autophagy, a cellular survival mechanism, by affecting cholesterol trafficking and mTOR signaling, which may sensitize cancer cells to other therapies and stress conditions [10,50,52]. The summary of experimental studies is given in Table 1. Please note that some studies include both experimental and clinical components; therefore, these studies are discussed together.

Multiple experimental investigations have highlighted itraconazole’s anticancer activity across diverse cancer models. In NSCLC, itraconazole was shown to inhibit angiogenesis and slow tumor growth in xenograft mouse models through suppression of endothelial cell functions and decreased tumor vascularization [11]. In another report, Zhang and colleagues demonstrated that itraconazole exerts potent anti-tumor effects in colorectal cancer (CRC) through its disruption of tumor energy metabolism. Specifically, in cell line-derived xenograft models using CRC-bearing mice, itraconazole significantly reduced tumor volume and weight by decreasing expression of CCAAT/Enhancer Binding Protein Beta (CEBPB), leading to suppression of enolase 1 (ENO1, a key glycolytic marker). Elevated CEBPB correlated with worse survival in CRC, underscoring the potential of itraconazole as a key therapeutic in cancer therapy [48]. Similarly, in colon cancer, itraconazole reduced cell viability by inducing cell cycle arrest, apoptosis, and autophagy, and was found to significantly improve five-year survival [50].

In head and neck squamous cell carcinoma (HNSCC) models, itraconazole has been found to inhibit Core 1 β1,3-galactosyltransferase (C1GALT1) [53]. C1GALT1 controls GalNAc-type-O-glycosylation and is overexpressed in several cancers. The overexpression of C1GALT1 leads to a poorer prognosis in cancer patients and is therefore an important target of anticancer therapies [53]. Itraconazole’s multifaceted mechanisms, including its effects on cancer cell proliferation, survival, and tumor microenvironment dynamics, highlight its promise in oncology.

### 4.2. Clinical Studies

As previously stated, several molecular mechanisms underlie itraconazole’s anticancer properties. One major mechanism is its ability to inhibit the Hedgehog (Hh) signaling pathway, which plays a critical role in the proliferation and survival of many cancer types [43,44,52]. Itraconazole has been shown to block Smoothened (Smo), a key component of the Hedgehog pathway and consequently tumorigenesis [43,54]. In cancer cells, Smo escapes its regulatory mechanism and is constitutively activated, leading to the transcription of GLI oncogenes that promote cell division and survival. Itraconazole’s ability to block Smo halts the abnormal activation that exists in tumor cells [43,54]. Combined with its well-established safety profile in humans and ability to be administered systemically, itraconazole’s unique ability to inhibit the Hedgehog signaling pathway makes it an ideal candidate for repurposing in cancer therapy [40,42,43]. To that end, a phase II randomized trial in men with metastatic castration-resistant prostate cancer found that high-dose itraconazole significantly delayed disease progression, likely via inhibition of the Hedgehog signaling pathway [7]. Researchers examined GLI1 expression in skin biopsies of the patients with prostate cancer, as Hh signaling is present in skin cells, and found that 11 of 40 patients experienced a twofold or greater downregulation in GLI1 with itraconazole treatment. Interestingly, the median PSA progression-free survival was significantly longer in patients with GLI1 downmodulation (*p* = 0.028) [7]. Similarly, in basal cell carcinoma, itraconazole suppressed tumor proliferation through inhibition of the Hedgehog pathway [55]. Itraconazole treated basal cell carcinoma patients experienced a 65% decrease in GLI1 mRNA (*p* = 0.028), with control group patients experiencing no significant decreases in GLI1 mRNA [55]. Notably, itraconazole has also been shown to block Smo independently of the canonical Smo-binding site, offering a novel therapeutic angle compared to traditional Hedgehog inhibitors [43]. The summary of clinical studies is given in Table 2.

Additionally, a phase II study on patients with biochemically recurrent prostate cancer evaluated the effects of itraconazole at a dose of 300 mg twice daily. The primary endpoint was a greater than or equal to 50% decline from baseline in serum prostate-specific antigen (PSA) after twelve weeks of itraconazole therapy. Although the cohort size was small (n = 19), nine patients (47%) achieved a decline in PSA by the end of twelve weeks, with one patient having a greater than 50% decline. This study demonstrated itraconazole’s ability to modulate serum PSA levels without altering testosterone concentrations. However, the observed effects were modest, and itraconazole treatment carried risks related to excess mineralocorticoid activity, including hypertension and hypokalemia [56].

Complementing these results, a window-of-opportunity study in NSCLC included thirteen patients scheduled for surgical resection who received itraconazole for ten to fourteen days preoperatively. The trial assessed the biological and pharmacodynamic effects of the drug. Itraconazole treatment resulted in reduced tumor volume, perfusion, pro-angiogenic cytokines, and microvessel density, indicating concentration-dependent anti-vascular, metabolic, and anti-tumor effects. The regimen was well-tolerated in all patients [57].

## 5. Immunotherapy and Anti-PD-1 Drugs

The immune system plays a significant role in the detection and destruction of cancer [58,59]. Not only can the immune system protect from virus-related cancers through the elimination of viral infections, but it can also reduce inflammation and recognize and eliminate malignant cells [59]. Therefore, preventing an inflammatory environment is important for preventing tumorigenesis, as chronic inflammation has been associated with the development of cancer [14,59]. Furthermore, the immune system can detect malignant cells through the expression of tumor-specific antigens [59]. After detection of malignant cells, several different types of immune cells, such as cytotoxic lymphocytes can kill the detected cancer cells [60,61]. These mechanisms highlight the importance of the immune system in hindering cancer development.

However, cancer cells can evade the immune response through several different mechanisms [14]. In some instances, cancer cells may lose their expression of tumor-specific antigens, allowing them to avoid recognition by immune cells [14]. For example, some NSCLCs have a loss of heterozygosity in human leukocyte antigens [14,62]. This leads to cancer cells presenting fewer antigens and avoiding detection by immune cells [14,62]. Another main component to the evasion of the immune response is the modulation of the TME, leading to an immunosuppressive TME [14]. Tumor growth is dependent on the TME, and secreting suppressive molecules and expressing inhibitory checkpoint molecules such as programmed cell death ligand 1 (PD-L1) allows tumors to undergo unmodulated growth [14,63].

Based on the involvement of the immune system in the mechanisms of cancer detection and eradication, immunotherapy has emerged as a prominent treatment for many cancer lines [64]. Immunotherapy is unique in that it utilizes the patient’s own immune system to identify and kill cancer cells [64]. One category of drugs commonly used in immunotherapy is anti-programmed cell death protein 1 (anti-PD-1) drugs, such as pembrolizumab and nivolumab [65,66]. Pembrolizumab and nivolumab were the first anti-PD-1 drugs approved by the FDA for the treatment of advanced melanoma [65,66]. Now, pembrolizumab and nivolumab are used in the treatment of NSCLC, head and neck squamous cell cancer, urothelial cancer, classical Hodgkin lymphoma, and renal cell cancer, liver cancer, bladder cancer, cervical cancer and uterine cancer [66,67,68].

**Figure 2:**
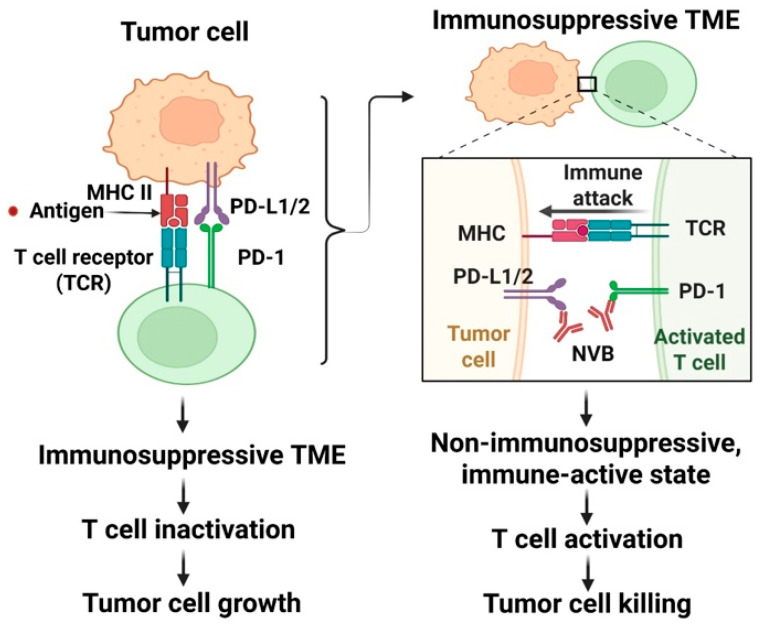
Schematic representation of the mechanism of anti-PD-1 immunotherapy. Antigenic peptides are presented via MHC class II molecules expressed on tumor cells. In addition, tumor cells express PD-L1 and PD-L2 proteins, which serve as ligands for the PD-1 receptor. Binding of PD-L1/PD-L2 to PD-1 creates an immunosuppressive tumor microenvironment, resulting in T-cell inactivation and tumor cell growth. Conversely, anti-PD-1 agents (e.g., nivolumab) disrupt the interaction between PD-L1/PD-L2 and PD-1, converting the immunosuppressive tumor microenvironment into an immune-active state that promotes T-cell activation and tumor cell eradication. Major Histocompatibility Complex, MHC; programmed cell death ligand 1 and 2, PD-L1/2; programmed cell death protein 1, PD-1; nivolumab, NVB.

Anti-PD-1 drugs target tumor-induced immune suppression [66]. Under normal conditions, PD-1 and PD-L1 function to maintain peripheral immune tolerance, so the immune system does not attack the body’s own cells [69]. PD-1 is expressed on a variety of cells in the body’s immune system, and PD-L1 is expressed on cancer cells and antigen-presenting cells [65,66]. In the TME, PD-1 and PD-L1 are attached as a receptor-ligand system, blocking the anti-tumor immune responses by T cells [65,66]. In some cancers, such as melanoma, PD-L1 is overexpressed, allowing tumor cells to effectively evade the immune response [66]. Therefore, anti-PD-1 drugs have emerged as an important component of immunotherapy due to their efficacy, low toxicity, and long-lasting response [69]. The mechanism of anti-PD-1 drugs is shown in Figure 2.

## 6. Combination Therapy

Combination therapy has been extremely beneficial for cancer treatment. It involves combining two or more therapeutic agents, which can enhance drug efficacy and reduce drug resistance while providing additional anticancer effects, such as the reduction in tumor growth and induction of apoptosis [70,71]. Typically, combination therapy utilizes drugs that target different cellular pathways to create a synergistic effect [70]. Itraconazole and anti-PD-1 drugs can be combined, as they target different cell pathways and lead to a synergistic effect [49]. Mechanistically, itraconazole can modulate the TME, blocking core 1 synthase and glycoprotein-N-acetylgalactosamine 3-beta-galactosyltransferase 1 (C1GALT1)-mediated O-glycosylation that leads to tumor-mediated immune evasion, enhancing the immune response of anti-PD-1 drugs [49].

Not only does itraconazole enhance the immune response to anti-PD-1 drugs, but combination therapy can help overcome drug resistance [70]. Despite immunotherapy becoming a cornerstone of cancer treatment, some patients may not respond to immunotherapy alone [72]. A major challenge of immunotherapy is determining which patients will respond to immunotherapy due to the heterogeneity of cancer and the capability of tumor cells to evade immune responses [72,73]. Researchers have focused on the combination of anti-PD-1 drugs with a second drug that targets other molecular pathways to help overcome drug resistance [73]. Combining itraconazole with anti-PD-1 drugs may help alleviate some of the drug resistance developed in patients undergoing cancer therapy.

Similarly, combining itraconazole with established chemotherapeutic regimens may help to overcome drug resistance and improve patient outcomes. As discussed above, itraconazole exerts anticancer effects through multiple complementary mechanisms, including inhibition of angiogenesis, disruption of tumor metabolism, and suppression of signaling pathways [11,43,44,52]. These mechanisms directly address several pathways implicated in chemotherapy resistance. When used in combination with the platinum-taxane chemotherapeutic regimen, itraconazole may enhance treatment efficacy by sensitizing tumor cells to cytotoxic injury [74]. Platinum agents induce DNA damage, while taxanes disrupt mitotic spindle formation and promote apoptotic cell death [19]. Itraconazole may potentiate these effects by impairing tumor cell survival signaling, reducing vascular support, and limiting adaptive metabolic responses. Through these synergistic actions, itraconazole has the potential to increase tumor cell susceptibility to chemotherapy, promote greater tumor cell death, and improve outcomes.

### 6.1. Experimental Studies Supporting Itraconazole’s Use in Combination Therapy

**Figure 3:**
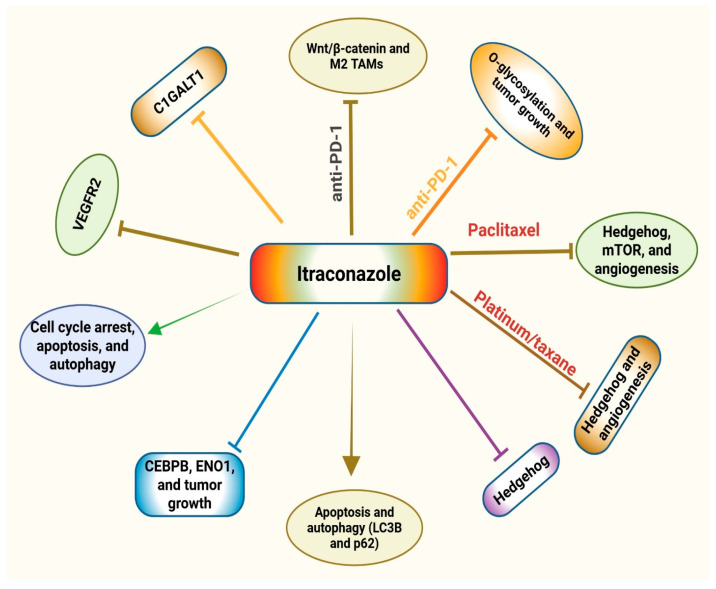
Schematic representation of the mechanisms of itraconazole alone and its combination with chemotherapy and anti-PD-1 immunotherapy. Itraconazole targets multiple signaling cascades to inhibit the growth of tumor cells via inducing cell cycle arrest, autophagy, and apoptosis. Itraconazole combination with chemotherapy and anti-PD-1 therapy results in the inhibition of multiple signaling mechanisms, leading to decreased angiogenesis and tumor growth. Core 1 β1,3-galactosyltransferase, C1GAT1; vascular endothelial growth factor receptor 2, VEGFR2; CCAAT/Enhancer Binding Protein Beta, CEBPB; enolase 1, ENO1; tumor-associated macrophages, TAMs; mammalian target of rapamycin, mTOR; Wnt/β-catenin pathway.

As the TME influences tumor growth via modulating various cellular components/activities, studies by Lin and colleagues determined the impact of targeting O-glycosylation-mediated immune cell crosstalk on the efficacy of anti-PD-1 immunotherapy in head and neck cancer (HNC) models. The data demonstrated that CRISPR/Cas9-mediated deletion of C1GALT1 in HNC cells resulted in tumor growth suppression in a syngeneic mouse model. Mechanistically, truncation of C1GALT1-induced O-glycosylation in HNC cells was found to promote M1 differentiation of macrophages, augmented cytotoxic T lymphocyte (CTL) activation/cytotoxicity, and decreased interleukin 6 (IL-6) levels. The combination of the O-glycosylation inhibitor, itraconazole, with anti-PD-1 therapy effectively suppressed tumor growth. Overall, the findings indicate that O-glycosylation targeting by itraconazole modifies TME to pro-inflammatory phenotypes, which augments the effectiveness of anti-PD-1 therapy [49]. A summary of the experimental evidence on the effects of itraconazole in combination with anti-PD-1 or chemotherapy is presented in Table 3. The schematic representation of the mechanisms of itraconazole alone and its combination with chemotherapy and anti-PD-1 immunotherapy is shown in Figure 3.

Along similar lines, Guan and colleagues evaluated the effects of the itraconazole and anti-PD-1 combination using the Ishikawa endometrial cancer cell (EC) model [75]. The data demonstrated that itraconazole alone inhibited the proliferation and invasion of Ishikawa cells in a dose- and time-dependent manner via inducing apoptosis. In combination with anti-PD-1, itraconazole significantly inhibited tumor invasion, which was mediated via increased secretion of IFN-γ, conversion of the M2 phenotype of tumor-associated macrophages (TAMs) into the M1 phenotype, and decreased secretion of interleukin 10 (IL-10) [75]. Mechanistically, itraconazole treatment resulted in decreased levels of Wnt-3a and β-catenin as well as increased expression of Axin-1, causing inhibition of Wnt/β-catenin signaling in TAMs. The in vivo studies in BALB/c nude mice complemented the in vitro findings in that synergistically decreased tumor volume, M2 to M1 polarization of TAMs, and inhibition of Wnt/β-catenin signaling were observed by the itraconazole and anti-PD-1 combination as compared to individual treatments [75]. These findings highlight the potential of itraconazole as a promising adjuvant to immune checkpoint inhibitors and warrant clinical investigation to further evaluate its therapeutic potential in human malignancies, including endometrial cancer.

In epithelial ovarian cancer, preclinical studies demonstrated that itraconazole enhanced the antitumor efficacy of paclitaxel in both in vitro and in vivo models. Combination therapy markedly reduced tumor weights compared to paclitaxel alone and was associated with lower microvessel density and reduced expression of angiogenesis, Hedgehog, and mTOR pathway markers [46].

Furthermore, researchers used Taiwan’s National Health Insurance database to find that colon cancer patients who received itraconazole had improved five-year survival, particularly in advanced disease (n = 5221) [50]. In models using two human colon cancer cell lines, treatment with varying concentrations of itraconazole was found to reduce viability in a concentration-dependent manner. Furthermore, it increased expressions of cleaved caspase-3 and Bax, promoted G1 cell cycle arrest, and elevated the proportion of sub-G1 cells, resulting in the induction of apoptosis. Itraconazole treatment also activated autophagy through LC3B activation and p62 involvement [50]. Such findings provide a strong biological rationale for investigating the combination of itraconazole and chemotherapy or anti-PD-1 therapy in human subjects. The summary of experimental studies with itraconazole in combination with immunotherapy or chemotherapy is given in Table 3.

### 6.2. Clinical Studies Supporting Itraconazole’s Use in Combination Therapy

Several clinical studies have since explored the potential benefits of combination therapy with itraconazole in cancer treatment [42,57,76,77]. Although large-scale randomized trials are currently lacking, smaller clinical trials and retrospective analyses have reported encouraging outcomes, particularly in patients with treatment-refractory solid tumors [8,42,56,57,76]. These studies often highlight improved progression-free survival and higher response rates compared to chemotherapy without combination therapy [8,56].

In a randomized controlled trial of patients with NSCLC, participants received platinum-based chemotherapy with or without itraconazole for a maximum of six cycles (n = 60). The primary outcome was one-year progression-free survival (PFS), with secondary endpoints including overall response rate, one-year overall survival, and tolerability. The itraconazole group achieved an overall response rate of 90% compared with 66.7% in the control group (*p* = 0.028). Mean one-year PFS was significantly improved with itraconazole, 6.56 months versus 5.415 months in the control group (*p* = 0.002). Adverse effects were generally well-tolerated, although one patient developed cardiotoxicity attributed to itraconazole [8].

A phase II clinical trial investigated the efficacy of combining itraconazole with pemetrexed in patients with progressive non-squamous NSCLC who had received one prior cytotoxic therapy. Participants were randomized to receive pemetrexed 500 mg/m^2^ with or without 200 mg of itraconazole daily for 21 days. Outcomes assessed included 3-month progression-free survival, median progression-free survival, overall survival, and toxicity. Among the 23 enrolled patients, 67% in the combination group were progression-free at three months versus 29% in the pemetrexed-only group (*p* = 0.11). Median progression-free survival was 5.5 months in the combination group compared to 2.8 months in the control group (*p* = 0.089), and overall survival was significantly longer in patients with itraconazole (32 months vs. 8 months, *p* = 0.012). Importantly, itraconazole was well tolerated, with no significant differences in toxicity between treatment groups [77]. Consistent with these findings, a retrospective clinical study reported improved survival among patients with refractory ovarian cancer who received itraconazole alongside chemotherapy compared to chemotherapy alone [78].

A notable case report of itraconazole treatment in a patient with primary malignant melanoma of the vagina provides mechanistic support for its integration with anti-PD-1 therapy. Itraconazole monotherapy was associated with rapid symptomatic improvement, radiographic tumor suppression, and transcriptional downregulation of tumor-promoting genes. Importantly, the patient exhibited disease progression and immune-related toxicity after initiating nivolumab alone but saw clinical stabilization upon reintroduction of itraconazole [79]. These findings raise the possibility that itraconazole may modulate the TME in ways that complement and enhance the efficacy of immunotherapy. By influencing angiogenesis, metabolic activity, and gene expression, itraconazole could help overcome resistance mechanisms to PD-1 inhibition, particularly in aggressive or immune-cold tumors [11,43,51,52]. This case highlights the need to explore itraconazole as a synergistic adjunct in immune checkpoint-based regimens. The summary of the clinical studies with itraconazole and chemotherapy is given in Table 4.

The rationale for combining immune checkpoint inhibitors and anti-fungal drugs for cancer treatment came from earlier studies demonstrating the activation of immune checkpoint molecules such as PD-1 during invasive yeast infections [80]. Patients with candida sepsis or candidiasis often experience heightened anti-inflammatory or immunosuppressive responses accompanied by impaired lymphocytes and phagocytes, T-cell exhaustion, and increased expression of PD-1 and PD-L1 on circulating T cells and NK cells [81,82,83,84]. In addition, co-induction of negative co-stimulatory molecules, PD-1, lymphocyte-activation gene-3 (LAG-3), and T-cell immunoglobulin and mucin domain-containing protein 3 (TIM-3) have been identified on peripheral blood mononuclear cells (PBMCs) of candida sepsis or candidiasis patients [81,82,83,84]. Consistent with these findings, blockade of immune checkpoint molecules, including PD-1, has resulted in improved survival in primary and secondary fungal sepsis [85,86]. As therapeutic modalities, including chemotherapy, can decrease anti-tumor immunity, resulting in infection or sepsis in cancer patients and contributing to an increased incidence of comorbidity and mortality [87,88], interventions with antifungals such as itraconazole and immune checkpoint inhibitors such as anti-PD-1 represent improved therapeutic strategies for these patients.

Despite the promise of combining itraconazole with chemotherapy or anti-PD-1 agents, several challenges must be addressed. One major concern is the potential for drug–drug interactions. Itraconazole is a potent inhibitor of cytochrome P450 enzymes, particularly CYP3A4, which may alter the metabolism of concurrent medications, including immunotherapies or supportive treatments, thereby increasing systemic drug exposure and the risk of toxicity [89,90]. In prior clinical studies, itraconazole was generally well tolerated, but adverse effects such as fatigue, nausea, elevated transaminase levels, hypertension, hypokalemia, and edema were observed, largely mild in severity and reversible [56,57]. Occasional hematologic toxicities were noted, but this was likely due to standard chemotherapeutic measures [77]. One patient in a combination trial of itraconazole with standard platinum-based chemotherapy developed cardiotoxicity, and the addition of itraconazole was discounted. However, this adverse event was rare [8].

Furthermore, both itraconazole and immune checkpoint inhibitors are independently associated with adverse effect profiles that can overlap or potentiate one another [40,67]. The addition of itraconazole may increase the incidence or severity of immune-related adverse effects such as hepatotoxicity, pneumonitis, or colitis [40,67]. To mitigate these risks, patients receiving combination therapy would require close clinical and laboratory monitoring, including routine liver function tests, pulmonary assessment for early signs of pneumonitis, and evaluation for gastrointestinal symptoms suggestive of colitis. Dose adjustments of itraconazole or temporary interruption of immunotherapy or chemotherapy may be necessary if significant toxicities arise. Additionally, interdisciplinary collaboration among oncologists, pharmacists, and other specialists is critical to balance therapeutic efficacy with patient safety, optimize drug dosing, and promptly manage emerging adverse effects during treatment.

## 7. Conclusions and Future Perspectives

Itraconazole shows strong potential as an anticancer agent, particularly when used in combination with chemotherapy or anti-PD-1 immunotherapy. By targeting multiple cellular pathways and modulating the tumor microenvironment, itraconazole not only impairs tumor growth but may also enhance the efficacy of immune checkpoint blockade [6,11,43]. This combination approach is especially valuable for overcoming resistance and addressing tumors that evade immune responses. By integrating itraconazole’s multifaceted mechanisms with the immune-activating properties of anti-PD-1 drugs, there is potential to create a synergistic effect that improves treatment efficacy across a broad spectrum of malignancies.

In parallel, antifungal drugs remain the cornerstone of therapy for invasive mycoses but are limited by toxicity, resistance, and suboptimal immune engagement [91]. Integrating immune checkpoint inhibitors with standard antifungal regimens could offer synergistic benefits: antifungal agents would directly reduce fungal burden, unmask immunogenic fungal epitopes, and potentially enhance antigen presentation, while checkpoint blockade would augment effector T-cell function and sustain antifungal immune responses [92].

Additionally, modulation of the host microbiota, including the mycobiome, by antifungal agents may influence systemic immune tone and responsiveness to checkpoint blockade, representing a novel mechanism for synergistic immunomodulation [92]. However, translation of these concepts into clinical practice faces several challenges, including the risk of immune-related toxicities, optimization of timing and dosing of combination therapy, and pathogen-specific differences in host immune responses. Future research should prioritize mechanistic investigations, rigorously controlled preclinical studies, and early-phase clinical trials to define the safety, efficacy, and biomarkers predictive of response to combined antifungal and immune checkpoint blockade therapy [93].

Taken together, the combinations of itraconazole with chemotherapy or anti-PD-1 immunotherapy have both demonstrated activity across various cancer types, reinforcing the clinical relevance of these therapeutic pairings. The repurposing of itraconazole offers an efficient path to translation, given its established safety profile and extensive history of use in humans. However, further investigation, including large-scale, randomized clinical trials, is essential to fully establish the therapeutic value and safety of this combination in oncology.

## Figures and Tables

**Table 1:** Summary of experimental studies on itraconazole for cancer.

Cancer Type	Study Type	Study Design and Patient Demographics	Key Results	Proposed Mechanism/Pathway	Refs
Non-Small Cell Lung Cancer	Xenograft models	NSCLC xenograft models derived from treatment-naïve patient tumors; itraconazole-treated cells compared with untreated controls	Itraconazole inhibited angiogenesis and slowed tumor growth	Suppression of endothelial cell function and reduced tumor vascularization	[11]
Advanced colorectal cancer	Itraconazole intraperitoneal injections	Xenograft models of CRC-bearing mice	Itraconazole significantly reduced tumor volume and weight	Itraconazole disrupts tumor energy metabolism by remodeling gene expression and cellular composition; it decreases expression of CEDPB and suppresses ENO1	[48]
Colon cancer	Clinical data and in vitro studies	Retrospective cohort of colon cancer patients treated or not treated with itraconazole; complementary in vitro assays on colon cancer cell lines	Itraconazole improved five-year survival and reduced cell viability and colony formation	Induction of cell cycle arrest, apoptosis, and autophagy	[50]
Head and neck squamous cell carcinoma	Mouse xenograft models and HNSCC cells	Using HNSCC cells and mouse xenograft models that overexpressed C1GALT1	Itraconazole significantly inhibited tumor growth and C1GALT1 expression	Itraconazole inhibits C1GALT1, a molecule that predicts poor prognosis in HNSCC	[53]

**Table 2:** Summary of clinical studies on itraconazole for cancer.

Cancer Type	Study Type	Study Design and Patient Demographics	Key Results	Proposed Mechanism/Pathway	Refs
Metastatic castration-resistant prostate cancer	Phase II clinical trial	Noncomparative randomized phase II trial in men with metastatic castration-resistant prostate cancer	High-dose itraconazole significantly delayed disease progression	Inhibition of the Hedgehog signaling pathway	[7]
Basal cell carcinoma	Open-label, proof-of-concept phase II trial	Patients with ≥1 basal cell carcinoma tumor > 4 mm in diameter received oral itraconazole 200 mg/day for 1 month or 100 mg twice daily for about 2 months	Itraconazole reduced tumor size, proliferation, and Hedgehog pathway activity	Inhibition of Hedgehog signaling	[55]
Biochemically recurrent prostate cancer	Phase II trial	Itraconazole 300 mg orally twice daily until PSA progression, toxicity, or patient withdrawal	47% of patients had a PSA decline by week 12; 5% had > 50% PSA reduction. Common adverse effects included edema, fatigue, hypertension, and hypokalemia	Inhibition of Hedgehog signaling	[56]
NSCLC patients scheduled for surgical resection	Phase 0, window-of-opportunity trial	Itraconazole 300 mg orally twice daily for 10–14 days before surgery	Decreased tumor perfusion and microvessel density; higher itraconazole levels correlated with greater tumor volume reduction and anti-angiogenic effects. Adverse effects were low-grade, reversible, and manageable, including fatigue, nausea, and elevated transaminase levels	Inhibition of Hedgehog signaling, alteration of tumor metabolism, inhibition of angiogenesis	[57]

**Table 3:** Summary of experimental studies on itraconazole in combination with immunotherapy or chemotherapy.

Cancer Type	Intervention	Cell/Animal Model	Key Results	Proposed Mechanism/Pathway	Refs
Head and neck cancer	Itraconazole combined with anti-PD-1 immunotherapy	Syngeneic mouse models of HNC	Combination therapy effectively suppressed tumor growth	Itraconazole inhibits O-glycosylation, enhancing immunosuppressive effects and augmenting the activity of anti-PD-1 therapy	[49]
Epithelial ovarian cancer	Itraconazole combined with paclitaxel	Ovarian cancer cell lines, mouse xenografts, and patient-derived xenograft models	Combination therapy reduced tumor weight, microvessel density, and angiogenesis more than paclitaxel alone	Inhibition of Hedgehog and mTOR signaling, anti-angiogenic effects	[46]
Colon adenocarcinoma	Itraconazole treatment; extends prior retrospective evidence that itraconazole improves 5-year survival in patients with late-stage colon cancer receiving chemotherapy	Human colon adenocarcinoma cell lines (COLO 205)	Itraconazole induced apoptosis and autophagy and promoted cell cycle arrest	Itraconazole increases cleaved caspase-3 and Bax expression and activates autophagy through LC3B activation and p62 involvement	[50]
Endometrial cancer	Itraconazole combined with anti-PD-1 immunotherapy	Syngeneic mouse model of EC	Combination therapy effectively suppressed tumor growth	Itraconazole and anti-PD-1 synergistically inhibited Wnt/β-catenin signaling and promoted the polarization of the M2 phenotype of TAMs to the M1 phenotype	[75]

**Table 4:** Summary of clinical studies with itraconazole and chemotherapy.

Patient Population	Design	Intervention	Outcomes	Limitations/Toxicities	Refs
Advanced NSCLC patients receiving platinum-based chemotherapy	Randomized controlled study	Addition of itraconazole to standard platinum-based chemotherapy vs. chemotherapy alone	1-year progression-free survival and 1-year overall survival were significantly improved in the itraconazole group	Itraconazole was well tolerated; one patient developed cardiotoxicity; the benefits were modest	[8]
Progressive non-squamous NSCLC after prior cytotoxic therapy	Phase II study	Pemetrexed IV with or without 200 mg oral itraconazole daily for 21 days	67% of patients were progression-free in the combination group vs. 29% with pemetrexed alone; both overall survival and median progression-free survival were longer with itraconazole	No significant differences in toxicities between groups	[77]
Refractory ovarian cancer	Retrospective study	Patients with refractory ovarian cancer receiving platinum/taxane chemotherapy with or without itraconazole	Itraconazole combination improved survival compared with chemotherapy alone	Synergistic effect with chemotherapy; potential Hedgehog and angiogenesis pathway inhibition	[78]

## Data Availability

No new data were created or analyzed in this study.

## Abbreviations

The following abbreviations are used in this manuscript:

ICs	Immune checkpoints
PD-1	Programmed cell death protein 1
PD-L1/2	Programmed cell death ligand 1/2
TME	Tumor microenvironment
FDA	Food and Drug Administration
HIV	Human immunodeficiency virus
mTOR	Mammalian target of rapamycin
NSCLC	Non-small-cell lung cancer
HNSCC	Head and neck squamous cell carcinoma
CRC	Colorectal cancer
CEBPB	CCAAT/Enhancer Binding Protein Beta
ENO1	Enolase 1
Hh	Hedgehog
Smo	Smoothened
VEGFR2	Vascular endothelial growth factor receptor 2
C1GALT1	Core 1 β1,3-galactosyltransferase
HNC	Head and neck cancer
CTL	Cytotoxic T lymphocyte
IL-6	Interleukin 6
PSA	Prostate-specific antigen
PFS	Progression-free survival
TAMs	Tumor-associated macrophages
LAG-3	Lymphocyte-activation gene-3
TIM-3	T-cell immunoglobulin and mucin domain-containing protein 3
PBMCs	Peripheral blood mononuclear cells
